# Improved Sugar Production by Optimizing Planetary Mill Pretreatment and Enzyme Hydrolysis Process

**DOI:** 10.1155/2015/267538

**Published:** 2015-10-11

**Authors:** Jeong Heo Kwon, Siseon Lee, Jae-Won Lee, Youn-Woo Hong, Jeong Ho Chang, Daekyung Sung, Sung Hyun Kim, Byoung-In Sang, Robert J. Mitchell, Jin Hyung Lee

**Affiliations:** ^1^Korea Institute of Ceramic Engineering and Technology (KICET), 101 Soho-ro, Jinju-si, Gyeongsangnam-do 52851, Republic of Korea; ^2^Department of Chemical Engineer, Hanyang University, Seoul 04763, Republic of Korea; ^3^School of Nano-Bioscience and Chemical Engineering, Ulsan National Institute of Science and Technology, Ulsan 44919, Republic of Korea; ^4^Department of Forest Products and Technology, Chonnam National University, Gwangju 61186, Republic of Korea

## Abstract

This paper describes an optimization of planetary mill pretreatment and saccharification processes for improving biosugar production. Pitch pine (*Pinus rigida*) wood sawdust waste was used as biomass feedstock and the process parameters optimized in this study were the buffering media, the milling time, the enzyme quantity, and the incubation time. Glucose yields were improved when acetate buffer was used rather than citrate buffer. Initially, with each process variable tests, the optimal values were 100 minutes of milling, an enzyme concentration of 16 FPU/g-biomass, and a 12-hour enzymatic hydrolysis. Typically, interactions between these experimental conditions and their effects on glucose production were next investigated using RSM. Glucose yields from the *Pinus rigida* waste exceeded 80% with several of the conditions tested, demonstrating that milling can be used to obtain high levels of glucose bioconversion from woody biomass for biorefinery purposes.

## 1. Introduction

Biofuel production is one of major emerging issues in environmental biotechnologies. Producing biofuels from biomass involve a series of processes: producing sugar from biomass through saccharification and biofuels from the sugar through fermentation in order of precedence. However, pretreatment of biomass, typically lignocellulosic, is a currently essential process to obtain high conversion of biomass prior to saccharification and fermentation because of the recalcitrance of biomass. Lignocellulosic biomass consists of cellulose, hemicelluloses, and lignin. Cellulose and hemicelluloses can be hydrolyzed to glucose and xylose, respectively, and subsequently converted to biofuels such as ethanol or butanol through fermentation. The purpose of pretreatment is to make the cellulose within the biomass more readily available for hydrolysis.

Previous study showed the potential of using planetary milling to pretreat biomass for downstream fermentation [1]. This method neither uses chemicals nor produces saccharification and fermentation inhibitors such as phenolics and furans, both of which are known to elicit stress responses within bacteria [2, 3]. Moreover, pretreatment with planetary milling does not involve high temperatures or pressures, making it more user friendly than many other pretreatment processes.

For an improved sugar production using planetary milling, several factors should be taken into consideration, including the milling time, the amount of enzyme added, and the time given for hydrolysis, as well as the type of buffer used during milling. Hence, we investigated the optimal conditions of pretreatment and enzymatic hydrolysis processes. Glucose yield is a result of combinational interaction between several experimental variables. Therefore, we investigated interactional effects of variable parameters during pretreatment and enzyme hydrolysis in this study, not only single parameter effects. We employed a softwood, pitch pine (*Pinus rigida*), as the feedstock for pretreatment and glucose production. Softwoods are dominant in the northern hemisphere but they have high lignin content and recalcitrant nature to enzymatic hydrolysis [4, 5], making them a model feedstock for this study.

## 2. Materials and Methods

### 2.1. Materials

The sawdust wood waste of pitch pine (*Pinus rigida*) after forestry manufacturing process was obtained from Suncheon Forestry Cooperative, Republic Korea. Compositional analysis of the sawdust wood waste was measured according to National Renewable Energy Laboratory protocols [6].

### 2.2. Planetary Mill Pretreatment

The planetary mill of sawdust wood waste of pitch pine (*Pinus rigida*) was carried out using a Pulverisette 5 (Fritsch, Germany) in zirconia jar container with 3 mm diameter zirconia balls. The jar was loaded with 7.2 g of biomass and 180 mL of 50 mM sodium acetate (pH 4.8) or 100 mM sodium citrate (pH 4.8). Planetary milling was performed at room temperature by changing the conditions of experimental factors such as milling time and types of buffer solution. The jar was rotated at a velocity of 300 rpm.

### 2.3. Enzymatic Hydrolysis

The buffer solutions used in planetary milling were suitable for cellulase activity. Hence, enzymatic hydrolysis process was continually carried out after the planetary milling without washing or exchanging buffer. A cellulase cocktail (Worthington Biochemical Co., USA) was added into the buffer containing milled biomass. Enzymatic hydrolysis was performed at 50°C in a shaking incubator (JEIO TECH, Rep Korea) at 200 rpm. The glucose yield was calculated using the following equation:

(1) Glucose  yield  %=Glucose  produced  by  enzyme  hydrolysisGlucan  in  sawdust  wood  waste×1.11×100

which was used in a previous study [7].

### 2.4. Characterization of the Samples

For investigating the morphology, pretreated pitch pine samples were incubated in dry oven. The samples were coated with Pt on a Cressington Scientific Instruments 108 Auto Sputter Coater (Cranberry Tep., USA). A scanning electron microscope (JSM-6700F, JEOL, Japan) was used to obtain microscopic images.

X-ray diffraction (Rigaku D/max-RB powder diffractometer, Japan) with Cu *κα* radiation (*λ* = 1.542 Å) was used for investigating the crystallinity of the samples. Samples were scanned at rate of 2°/min.

The quantities of glucose produced were measured by test strips (Glucose Test Method, Merck, Germany). The color changes were analyzed using a Reflectometer (RQflex plus 10, Merck, Germany). Test strips (Glucose Test Method, Merck, Germany) were sufficiently immersed into test solutions and placed in strip adapter to measure colorimetric changes. Finally, the software installed in the Reflectometer calculated the quantities of glucose contained in the sample by using colorimetric change.

### 2.5. Data Analysis

All experiments were performed in triplicate for error analysis. Minitab 14 (Minitab Inc., USA) was used for 3D surface plots.

## 3. Results and Discussion

Table 1 presents the composition of pitch pine sawdust used in this study. We used this softwood as a feed stock material to clearly investigate the effect of various conditions during pretreatment and saccharification since it is regarded as a high lignin-containing biomass having a strong structure [4, 5]. When compared with the previously reported composition for untreated* Pinus rigida* [8], some slight variations were seen. For example, the lignin content was somewhat higher in our samples, 33.13% as compared with 29%, while the hemicellulose portion was lower, that is, 20.13% versus 23.7%. The cellulose content of* P. rigida*, however, was similar in both studies with values slightly higher than 40%. The pitch pine sawdust waste used in study had a crystallinity index of 52.5% (Table 2). As this is higher than rice straw, which had a crystallinity index of 48% in our previous study [1], it suggests that the cellulose within* P. rigida* is more recalcitrant to treatment and hydrolysis than that of rice straw. Consequently, effects of the experimental conditions during pretreatment or saccharification can be more distinctly investigated with this biomass sample to improve glucose yield.

**Figure 1:**
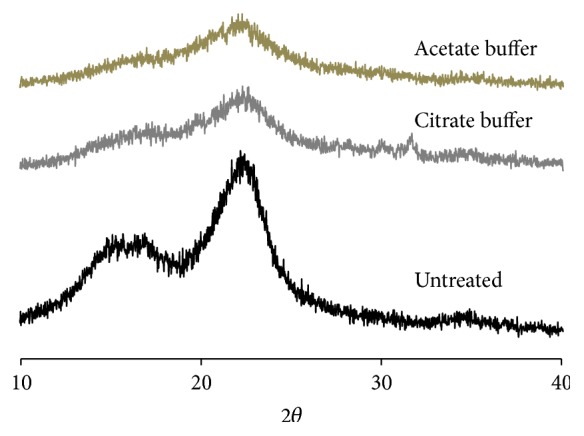
XRD patterns obtained for untreated pitch pine sawdust waste without planetary milling and for the milled samples using either an acetate or citrate buffer.

**Figure 2:**
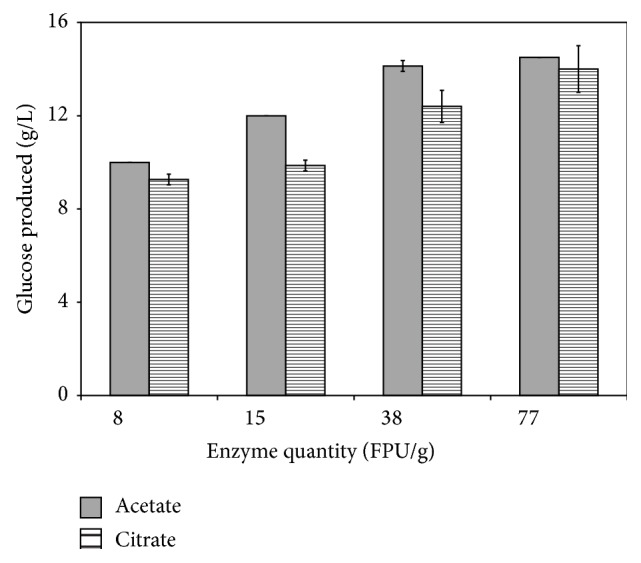
Comparison of the glucose yields obtained from the acetate and citrate buffers. Glucose was produced under the following conditions: 6 hr milling and 24 hr incubation. The quantities of the enzymes used are listed and were 8, 15, 38, and 77 FPU/g, respectively.

Acetate and citrate buffers are commonly employed during the saccharification of plant-based cellulose using enzymes to produce glucose [1, 8, 9]. To minimize the number of procedural steps required and their impact on glucose yields, including the loss of biomass, we chose to evaluate these two buffers and their effects within planetary milling processes. Pretreatment of plant biomass by planetary milling was shown previously to reduce the crystallinity of the sample [1].  Figure 1 shows the X-ray diffraction (XRD) patterns for the untreated pitch pine sample and after planetary milling in either sodium citrate or sodium acetate solution. There are two important peaks on XRD spectrums of lignocellulosic biomass: the peak at 18.7°, which represents only amorphous cellulose, and that at 22.5°, which corresponds to both crystalline and amorphous cellulose. As shown in Figure 1, the intensities of the 22.5° peaks were drastically reduced in the pretreated samples after only 20 minutes of milling when either the acetate or citrate buffer was used. According to “crystallinity index,” which is a ratio of crystalline cellulose to amorphous cellulose content and is defined as (*I*_2*θ*=22.5°_ − *I*_2*θ*=18.7°_)/(*I*_2*θ*=22.5°_) × 100, planetary milling was very effective in reducing the crystallinity of the* P. rigida *cellulose. The crystallinity index of the untreated pitch pine sawdust was 52.5% but was reduced to 36.5 and 31.7% only after 20 minutes of planetary milling when citrate or acetate buffers were used, respectively (Table 2). This represents a reduction of 30% and 40% for the two buffers, respectively, indicating that acetate buffer leads to a greater level of decrystallization. This finding was further supported by the glucose yields that resulted after enzymatic treatment of the milled biomass within the respective buffers (Figure 2). As shown in this figure, glucose yields improved as the concentration of each treated biomass increased but treatment of the biomass milled in the acetate buffer consistently gave better yields. Hence, we found that acetate buffer was more effective to be employed in both planetary milling pretreatment and enzymatic saccharification.

**Figure 3:**
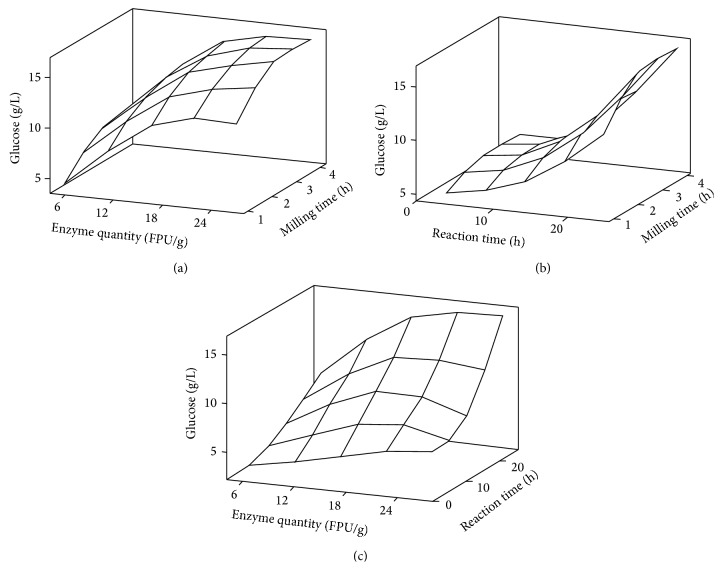
Combined impacts of the parameters on the resulting glucose concentrations. In each case, one of the variables was locked and the other two were varied. The plots show the relationship between and effects of the (a) milling time and enzyme quantity, (b) milling time and enzyme reaction time, and (c) enzyme reaction time and enzyme quantity. The standard values for the locked parameters were milling for 4 hours, 27 FPU/g-biomass, and an enzymatic hydrolysis time of 24 hours.

The effects of experimental conditions on the glucose yield are combinational through the interactions between experimental variables. Initially, we investigated the effects three single variable parameters, that is, milling time, enzyme quantity, and reaction time, had on the glucose yields and found optimal values at 100 minutes, 16 FPU/g-biomass, and 12 hours, respectively (see Supplementary Material available online at http://dx.doi.org/10.1155/2015/267538). To investigate interactional effects of variable parameters, a combinatorial chart was prepared using the conditions listed above as base values along with higher and lower parameter values for analysis (Table 3). When the enzyme reaction time was set for 24 hours, it is clear that the enzyme quantity is a more dominant factor controlling the glucose yields than the milling time (Figure 3(a)). Moreover, the improvement in the glucose yields as greater amounts of enzyme were added was consistent, regardless of the milling time. This is clearly presented in Table 3 where the percent yields under these conditions increased by 35.2% to 37% for the 2-hour and 4-hour milling times, respectively, when the enzyme concentrations were increased from 5 FPU/g-biomass to 16 FPU/g-biomass. Likewise, the use of 27 FPU/g-biomass with a 24-hour reaction time led to average increase in glucose yields of 43.8% when compared to the same experiments performed with 5 FPU/g-biomass. This consistent improvement in the glucose yields suggests that the cellulose is readily available for hydrolysis by the enzymes and that the bottleneck limiting the yields lies more with the enzyme availability. Another factor that should be considered, when trying to explain why the greater addition of enzyme leads to better yields, is the accessibility of cellulose during the saccharification process. During this process, cellulase tends to irreversibly bind to the hydrophobic surface of biomass and loses it activity [10]. Also cellulase could be buried within the fibrillar architecture of the cellulose microfibrils [11]. Both of these processes work to reduce the effective activity of the enzymes but can be overcome by larger enzyme additions.

Performing similar experiments but with the cellulase enzyme concentration locked at 27 FPU/g-biomass, we once more found that the milling time did not influence the glucose yields as much as the hydrolytic treatment times, since longer reaction times give significantly better glucose yields. Interestingly, the yields did not increase linearly but rather in an exponential fashion with longer treatments (Figure 3(b)). As shown in Table 3, a comparison between the different reaction times used found that12 hours improved the yields on average by about 1.23-fold over a 3-hour treatment while the yields after a 24-hour treatment were 2-fold higher than the 12-hour treatment. This enhanced productivity over time was initially thought to be due to the early stage hydrolysis of the cellulose by the enzymes as this would improve access to the cellulose. However, as shown in Figure 3(c), this trend was only seen when a high concentration of enzyme was used.

It is clear from this figure that both of these parameters are influential, as noted above, but the reaction time had a greater effect on the glucose yield. The loss in yield with higher enzyme additions is also a fortuitous finding as the cost of the enzyme used is one of the major contributors to biofuel production costs. One recent study stated that the costs incurred by the use of these enzymes have been significantly underestimated by a large number of groups and their studies [12]. As such, the finding that less enzyme gives more glucose is encouraging, even though this study shows that with longer reaction times this benefit is lost. Table 3 shows that three of the parameter groupings lead to glucose yields that exceed 80% of the expected maximum yield. All three required a 24-hour enzymatic treatment time but varied with respect to the milling time and the enzyme concentration. While an enzyme addition of 27 FPU/g-biomass is used within two of these, the percent yield with 16 FPU/g-biomass is comparable, implying that longer milling times can partially off-set the costs associated with the enzymes. One additional cost-related benefit offered by planetary milling is that this process does not involve any washing steps. Hence, we can obtain a 100% recovery of the biomass solids and sugars, which is very useful to the biorefinery industry [13, 14].

## 4. Conclusions

We have investigated the improvement way of glucose yield by optimizing the planetary milling pretreatment and enzymatic saccharification processes for* P. rigida *waste. Although milling effectively reduced the cellulose crystallinity in both acetate and citrate buffers, the results with acetate were slightly better and this presumably contributed to the higher glucose yields resulting with this buffer. By evaluating combinatorial parameter effects with RSM, we obtained glucose yields that surpassed 80% from* P. rigida *waste. Moreover, we demonstrated that the costs associated with the cellulase enzymes can potentially be mitigated by increasing the milling or enzymatic hydrolysis times.

## Supplementary Material

The effects of three single variable parameters on the glucose yields were investigated before measuring the combinational effects of experimental conditions. Figure S1 shows the effects each of the individual variables had on the glucose yields. As increasing the value of each variable, the productions of glucose were increased and reached to saturation points. Finally, optimal values for single variable were found at 100 minutes, 16FPU/g-biomass, and 12 hours for milling time, enzyme quantity and reaction time, respectively (Fig S1). The standard values for the locked parameters were milling for 8 hours, 42.5 FPU/g-biomass and a enzymatic hydrolysis time of 72 hours.
After planetary milling, the sizes of biomass were reduced. However, there was no significant difference of biomass size depending on milling time. When it compared with non-pretreated sample, it was clear that planetary mill disrupted the rigid surface and its intact phytoliths (Fig S2). Figure S2 shows scanning electron micrographs of before (a) and after 40 (b), 60 (c) and 120min (d) planetary milled pitch pine wood wastes. Small pieces of non-milling sample were selected to obtain SEM image. Even though the size of biomass does not homogenous, planetary mill was effective to reduce biomass size.

## Figures and Tables

**Table 1:** Absolute compositional percentage of each component within the native pitch pine (*Pinus rigida*).

Cellulose Glucan (%)	Hemicellulose				Lignin		Acetyl (%)	Ash (%)
	Xylan (%)	Arabinan (%)	Galactan (%)	Mannan (%)	Acid-insoluble (%)	Acid-soluble (%)		
41.54 ± 0.27	5.75 ± 0.66	2.66 ± 0.08	2.2 ± 0.02	9.52 ± 0.02	32.35 ± 0.11	0.78 ± 0.04	0.83 ± 0.04	0.37 ± 0.02

**Table 2:** Comparison of the pitch pine sawdust waste crystallinity after 20 minutes of planetary milling in an acetate or citrate solution.

	Untreated	Acetate	Citrate
Crystallinity index (%)	52.5 ± 0.9	31.7 ± 0.3	36.5 ± 5.1
Relative crystallinity index^1^	1.00 ± 0.02	0.60 ± 0.01	0.70 ± 0.10

^1^Relative crystallinity index: crystallinity index after planetary milling/crystallinity index of untreated sample.

**Table 3:** Experimental parameters used in the response surface methodology and their effects on the glucose yield. The parameter values that led to greater than yield that exceeded 80% are shown in bold.

Enzyme quantity (FPU/g-biomass)	Enzyme reaction time (hr)	Milling time (hr)	Glucose yield (%)
5	3	1	12.7 ± 1.2
5	3	2	14.3 ± 0.2
5	3	4	16.4 ± 0.6
5	12	1	16.8 ± 0.5
5	12	2	20.2 ± 3.0
5	12	4	25.7 ± 0.8
5	24	1	23.5 ± 0.8
5	24	2	36.7 ± 3.2
5	24	4	44.7 ± 1.4
16	3	1	22.1 ± 0.8
16	3	2	26.2 ± 0.3
16	3	4	26.9 ± 0.3
16	12	1	36.3 ± 1.4
16	12	2	40.0 ± 2.4
16	12	4	48.3 ± 0.5
16	24	1	59.6 ± 1.1
16	24	2	71.9 ± 7.2
**16**	**24**	**4**	**81.7 ± 4.1**
27	3	1	27.5 ± 1.3
27	3	2	33.8 ± 2.4
27	3	4	35.4 ± 1.9
27	12	1	35.4 ± 2.7
27	12	2	42.5 ± 1.4
27	12	4	40.5 ± 9.2
27	24	1	66.2 ± 4.7
**27**	**24**	**2**	**82.8 ± 0.6**
**27**	**24**	**4**	**87.5 ± 1.2**
