# Supplementary material for: Improved Sugar Production by Optimizing Planetary Mill Pretreatment and Enzyme Hydrolysis Process
Source: Biomed Res Int. 2015 Oct 11;2015:267538. doi: 10.1155/2015/267538 (PMC4619780; doi:10.1155/2015/267538)
Supplement: Supplementary file 1 — The effects of three single variable parameters on the glucose yields were investigated before measuring the combinational effects of experimental conditions. Figure S1 shows the effects each of the individual variables had on the glucose yields. As increasing the value of each variable, the productions of glucose were increased and reached to saturation points. Finally, optimal values for single variable were found at 100 minutes, 16FPU/g-biomass, and 12 hours for milling time, enzyme quantity and reaction time, respectively (Fig S1). The standard values for the locked parameters were milling for 8 hours, 42.5 FPU/g-biomass and a enzymatic hydrolysis time of 72 hours. After planetary milling, the sizes of biomass were reduced. However, there was no significant difference of biomass size depending on milling time. When it compared with non-pretreated sample, it was clear that planetary mill disrupted the rigid surface and its intact phytoliths (Fig S2). Figure S2 shows scanning electron micrographs of before (a) and after 40 (b), 60 (c) and 120min (d) planetary milled pitch pine wood wastes. Small pieces of non-milling sample were selected to obtain SEM image. Even though the size of biomass does not homogenous, planetary mill was effective to reduce biomass size. [file 267538.f1.pdf]

Before measuring the combinational effects of experimental conditions, the effects of three single variable parameters on the glucose yields were investigated. We found optimal values at 100 minutes, 16FPU/g-biomass, and 12 hours for milling time, enzyme quantity and reaction time, respectively (Fig S1).

(a)

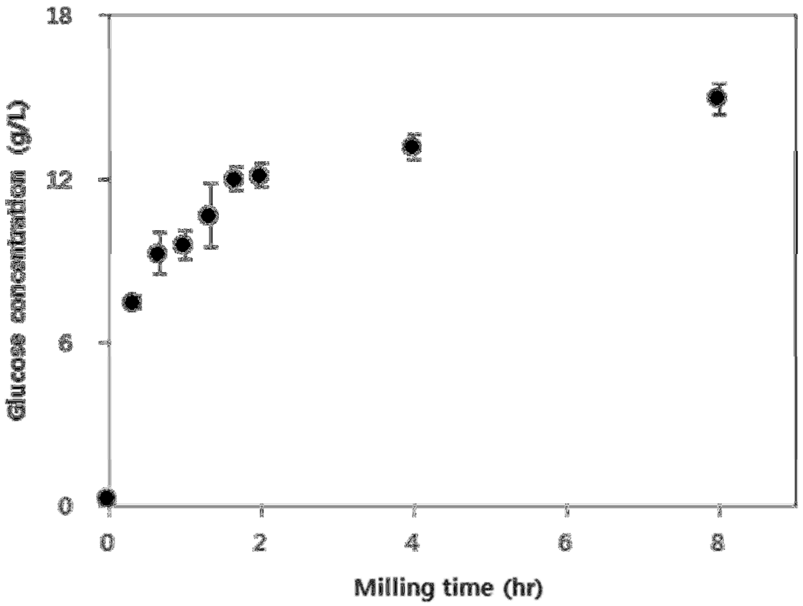

(b)

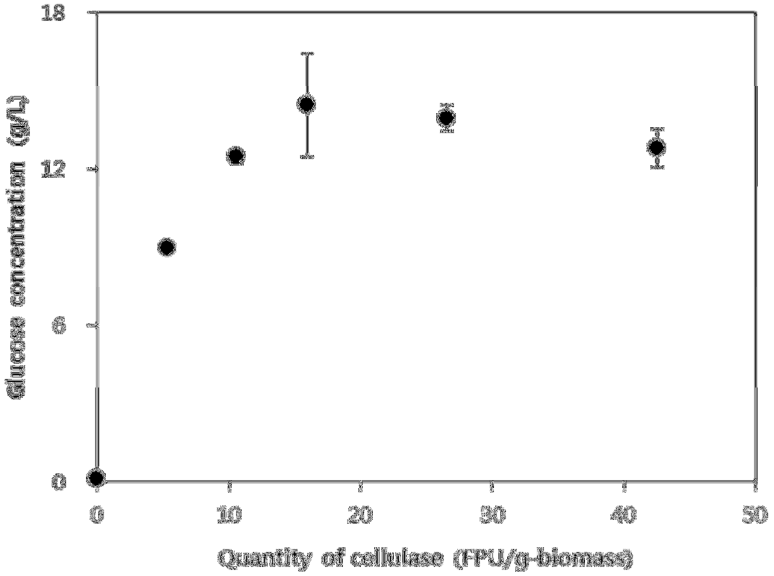

(c)

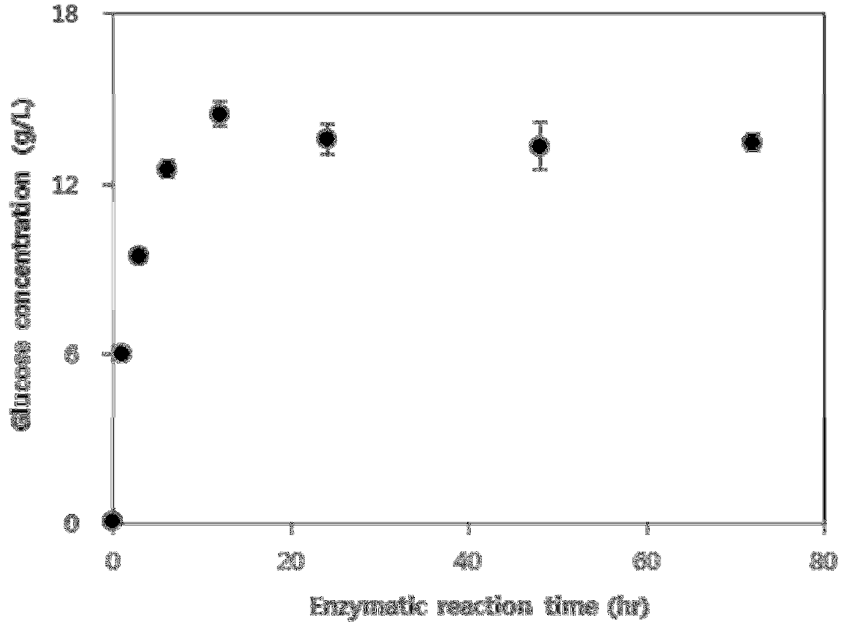

Fig S1. Plots showing the effects each of the individual variables had on the glucose yields. The variable tested were the (a) milling time, (b) quantity of cellulase added and (c) enzymatic reaction time. The standard values for the locked parameters were milling for 8 hours, 42.5 FPU/g-biomass and a enzymatic hydrolysis time of 72 hours.

After planetary milling, the sizes of biomass were reduced. However, there was no significant difference of biomass size depending on milling time. When it compared with non-pretreated sample, it was clear that planetary mill disrupted the rigid surface and its intact phytoliths (Fig S2)

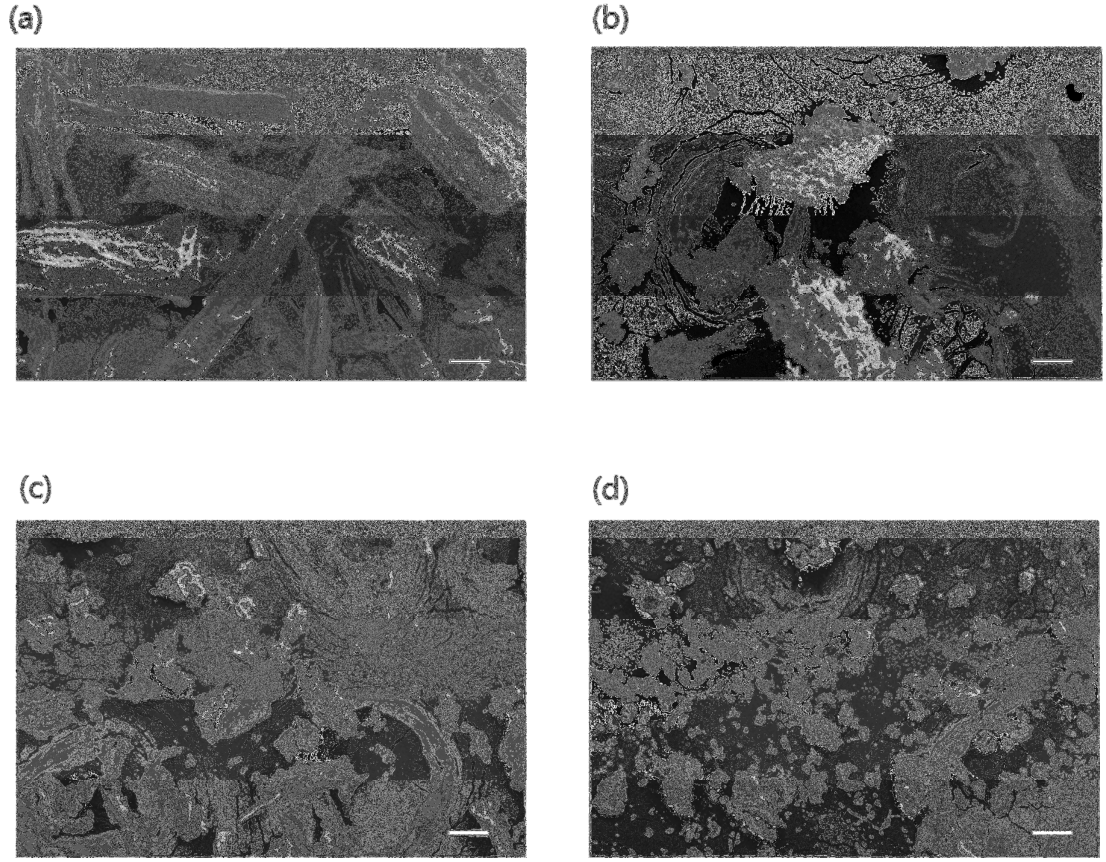

Fig S2. Scanning electron micrographs of before (a) and after 40 (b), 60 (c) and 120min (d) planetary milled pitch pine wood wastes. Small pieces of non-milling sample were selected to obtain SEM image. Even though the size of biomass does not homogenous, planetary mill was effective to reduce biomass size. The bar is 50 μm
